# Efficacy and safety of drug-coated balloon combined with cutting balloon for side branch of true coronary bifurcation lesions: Study protocol for a multicenter, prospective, randomized controlled trial

**DOI:** 10.3389/fcvm.2022.1035728

**Published:** 2022-11-03

**Authors:** Haoyu Wu, Jizhao Deng, Lei Liang, Xinjun Lei, Xiaowei Yao, Wenqi Han, Haichao Chen, Xiling Shou

**Affiliations:** ^1^Department of Cardiology, Shaanxi Provincial People's Hospital, Xi'an, China; ^2^Department of Cardiology, The First Affiliated Hospital of Xi'an Jiaotong University, Xi'an, China

**Keywords:** coronary bifurcation lesion, drug-coated balloon, cutting balloon, side branch, percutaneous coronary intervention, clinical trial

## Abstract

**Background:**

Coronary bifurcation lesions are common of percutaneous coronary intervention (PCI), and the optimal interventional therapy strategy is still a matter of debate and remains a challenge for interventional cardiologists. The provisional stenting technique is still a preferred method for most bifurcation lesions, but restenosis of the side branch (SB) occurs in approximately 17–19% of cases. Therefore, the dilemma of reducing SB restenosis still exists, and further research on strategies to reduce restenosis for SB is necessary. Drug-coated balloon (DCB) can reduce clinical events in small vessel disease and in-stent restenosis. The efficacy and safety of DCB for SB of true coronary bifurcation lesions have not been fully investigated. A randomized comparison of DCB combined with cutting balloon angioplasty vs. cutting balloon angioplasty for SB has never been published.

**Methods and design:**

The purpose of this study is to explore the superiority of DCB combined with cutting balloon vs. cutting balloon angioplasty for SB after main vessel (MV) drug-eluting stent implantation of true coronary bifurcation lesions. This study is a multicenter, prospective, randomized controlled trial including 140 patients with true coronary bifurcation lesions. Patients will be randomized in a 1:1 manner to receive either DCB combined with cutting balloon or cutting balloon angioplasty for SB after MV drug-eluting stent implantation. The primary endpoint is the evaluation of late lumen loss (LLL) of SB at the 9-month follow-up. The secondary endpoints include procedural success during initial hospitalization, LLL of MV at the 9-month follow-up, binary angiographic restenosis in MV and SB at the 9-month follow-up, the proportion of patients with a final post-PCI quantitative flow ratio result ≤ 0.80 for SB at the 9-month follow-up, and major adverse cardiac events during the 24-month follow-up.

**Conclusions:**

This clinical trial will provide evidence as to whether DCB combined with cutting balloon for SB of true coronary bifurcation lesions is a superior treatment approach.

**Trial Registration Number:**

ChiCTR2000040475.

**Dissemination:**

The results of this clinical trial will be published in a peer-reviewed journal.

## Introduction

Coronary bifurcation lesions are involved in 15–20% of cases of percutaneous coronary intervention (PCI), which is difficult for interventional cardiologists to address (1, 2). PCI for bifurcation lesions is a challenging technique and has more procedural complications, a higher restenosis rate and worse clinical outcomes (3, 4). The optimal interventional therapy strategy for bifurcation lesions remains controversial. The use of two-stent technique in some bifurcation lesions will result in substantial metal residues in the lumen, which is particularly relevant to stent thrombosis and appears not to lower the risk of restenosis (5). Provisional stenting remains the preferred approach for most bifurcation lesions, but regardless of whether there is a final kissing balloon inflation at the end, restenosis of the side branch (SB) occurs in approximately 17–19% of cases (6). Therefore, the dilemma of reducing SB restenosis still exists, and further research on strategies to reduce restenosis for SB in coronary bifurcation lesions is necessary.

Drug-coated balloon (DCB) allows the release of anti-proliferative agents to the vascular wall through a semi-compliant balloon without leaving any metal, which can inhibit the proliferation of vascular smooth muscle cells (7). DCB has been reported to have good efficacy and safety for in-stent restenosis and new-onset small vessel disease (8). DCB could also be a promising treatment modality for SB (5). The combined use of drug-eluting stent (DES) in the main vessel (MV) and DCB in the SB to treat coronary bifurcation lesions is attractive and may be accompanied by improved clinical outcomes (9). The BIOLUX-I (10) and DEBSIDE (11) clinical trials suggested that a DES in MV and a DCB in SB had low late lumen loss (LLL). The use of DCB in bifurcation lesions can maintain the simplicity of provisional stenting and reduce SB restenosis. However, existing studies exploring the efficacy and safety of DCB for SB in coronary bifurcation lesions have many limitations (for example, selection for SB and technology), and no conclusive evidence has been provided thus far (5).

Compared with traditional percutaneous transluminal coronary angioplasty, the benefits of cutting balloon angioplasty for plaque modification in bifurcation lesions have been demonstrated (12). The use of cutting balloon for SB in coronary bifurcation lesions can effectively prevent plaque displacement or plaque protruding into the MV, which can prevent complex two-stent technique and reduce the incidence of SB compromise and restenosis (13). Compared with cutting balloon angioplasty, the combination of DCB and cutting balloon for SB in coronary bifurcation lesions may be more effective and safer. However, to the best of our knowledge, no randomized trials have been performed to compare DCB combined with cutting balloon angioplasty vs. cutting balloon angioplasty for SB after a DES in MV of true coronary bifurcation lesions. Accordingly, the proposed multicenter, prospective, randomized controlled study is to explore the superiority of DCB combined with cutting balloon to cutting balloon angioplasty for SB after a DES in MV of true coronary bifurcation lesions.

## Methods and analysis

### Study hypothesis

The purpose of this trial is to investigate the hypothesis that DCB (paclitaxel-eluting balloon: Vesselin^®^) combined with cutting balloon is superior to cutting balloon angioplasty for SB after a DES in MV of true coronary bifurcation lesions at the 24-month follow-up.

### Study design

**Figure 1:**
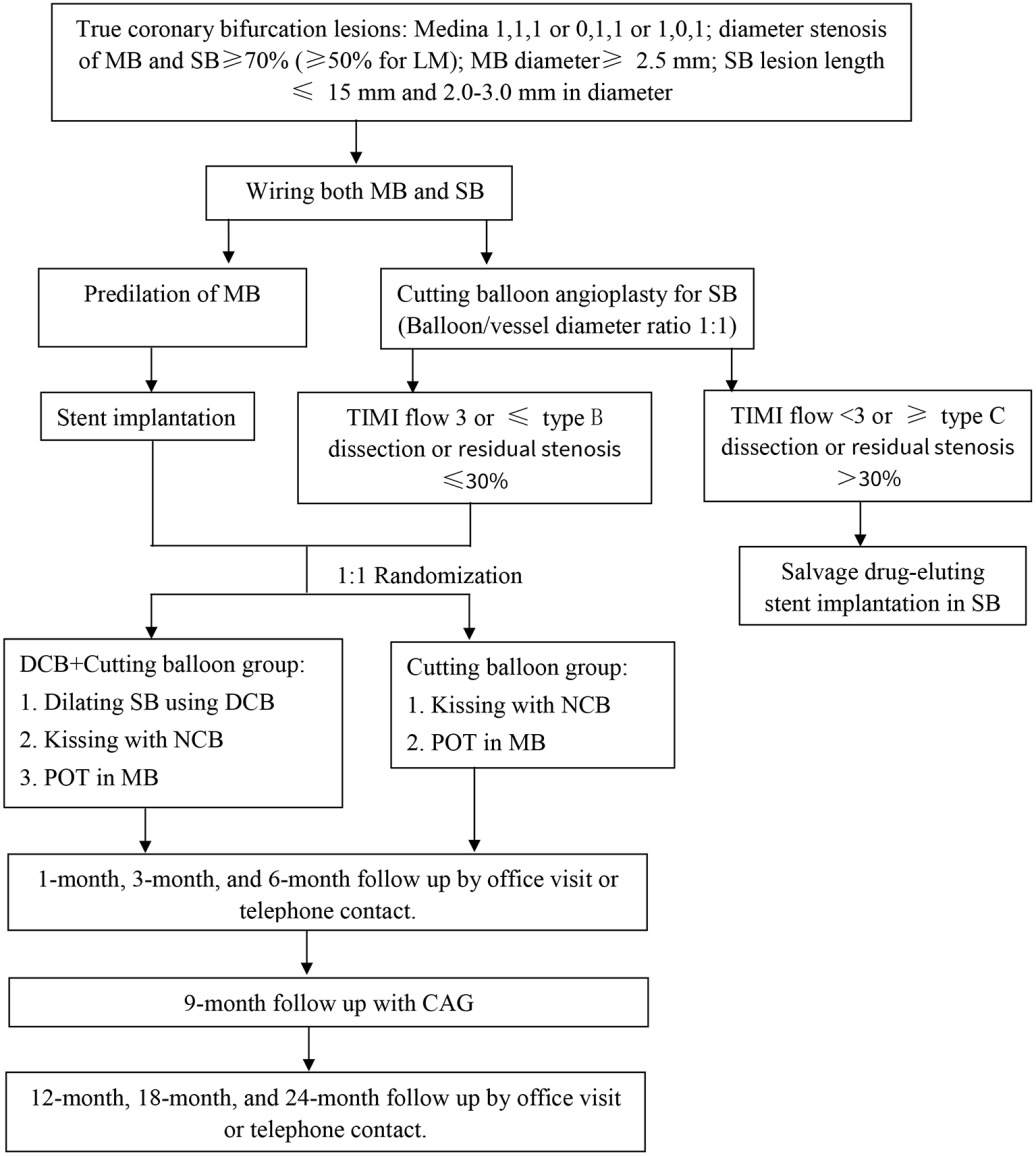
Flowchart of the clinical trial design. MB, main branch; SB, side branch; LM, left main; TIMI, thrombolysis and thrombin inhibition in myocardial infarction; DCB, drug-coated balloon; NCB, non-compliant balloon; POT, proximal optimization technique; CAG, coronary angiography.

This study is a multicenter, prospective, randomized controlled trial at 8 medical centers in China, including a total of 140 patients. The overall study flow chart is summarized in Figure 1. This clinical trial is conducted according to the Declaration of Helsinki. The clinical trial protocol and written informed consent forms were reviewed and approved by the Clinical Trial Ethics Committee of Shaanxi Provincial People's Hospital and each medical center. Written informed consent is required from the enrolled patients. The study protocol has been registered in the China Clinical Trial Registry (ChiCTR2000040475). The patient data in the Data Management System will be password-protected and available only to study-designated users with the appropriate authorization level. Although the operators are not blinded, all individuals who analyze the data will be blinded to the treatment strategy. Peer-reviewed journals will publish the results of this clinical trial, and international conferences will also disseminate the findings.

### Study population and randomization

A total of 140 patients with true coronary bifurcation lesions (Medina 1, 1, 1, or 0, 1, 1 or 1, 0, 1) are randomly divided into a research group (DCB combined with cutting balloon) or a control group (cutting balloon) for SB in a 1:1 manner after a DES in MV. The random sequence numbers of the patients will be produced by an Interactive Web Randomization System.

#### Inclusion criteria

(1) Over 18 years old.

(2) Subjects with silent ischemia, stable or unstable angina, or acute myocardial infarction over 1 week.

(3) The target lesion must be a true bifurcation lesion (Medina 1, 1, 1 or 0, 1, 1 or 1, 0, 1) and suitable for PCI. Lesions are defined according to the Medina classification (14).

(4) Reference vessel diameter of the target lesion: MV ≥ 2.5 mm and SB 2.0-3.0 mm.

(5) Preoperative vessel diameter stenosis of MV ≥ 70% (left main coronary artery stenosis ≥ 50%). Preoperative vessel diameter stenosis of SB ≥ 70%.

(6) Lesion length of SB ≤ 15 mm.

(7) If the target lesion is chronic total occlusion, the chronic total occlusion lesions of MV or SB should have been successfully recanalized before enrollment.

(8) If a non-target lesion requires PCI, the non-target lesion must undergo PCI first.

(9) The subject (or legal guardian) can understand the protocol.

(10) Subjects (or legal guardians) can provide written informed consent.

(11) The subject is willing to follow the protocol of the clinical trial.

#### Exclusion criteria

(1) Myocardial infarction within 1 week.

(2) Known allergy to the balloon/stent system or accompanying drugs in this study.

(3) Intolerance to dual antiplatelet therapy.

(4) Life expectancy < 24 months.

(5) Pregnancy or breastfeeding.

(6) The subject is participating in another trial.

(7) Congestive heart failure with left ventricular ejection fraction < 30% or NYHA class IV.

(8) Serum creatinine > 2.0 mg/dL (176.82 μmol/L).

(9) In-stent restenosis.

(10) The target lesion involves an aneurysm or is adjacent to an aneurysm (within 5 mm).

(11) Severe calcification requiring rotational atherectomy.

(12) Failure to follow the protocol or follow-up requirements.

(13) The target lesion is a bypass graft vessel.

### Study procedures

After wiring the MV and SB, the MV is fully predilated with balloons suitable for the length of the lesion and at an appropriate pressure. The SB is dilated with cutting balloon suitable for the length of the lesion and with an appropriate pressure (if the cutting balloon is difficult to pass, a predilated balloon that is easy to pass can be used for low-pressure dilation, and then the cutting balloon is used). The diameter ratio of the cutting balloon to vessel is 1:1. If there is TIMI flow < 3 or ≥ type C dissection or residual stenosis > 30% in SB, a systematic two-stent technique will be performed to treat the target lesions.

If there is TIMI flow 3, ≤ type B dissection and residual stenosis ≤ 30% in SB, a DES (1:1 according to the distal MV diameter) will be implanted in the MV across the SB with jailing of the SB wire. The wire will then be exchanged. The SB will be rewired through the stent struts (*via* a distal stent strut is preferred). Post-dilation will be performed using a non-complaint balloon to optimize the stent expansion (residual diameter stenosis < 10%). The strut of the stent at the opening of the SB can be expanded with a suitable balloon for any follow-up operations. Patients will be randomly divided into a research group (DCB combined with the cutting balloon) or a control group (cutting balloon) for SB in a 1:1 manner.

Research group: SB dilation will use a paclitaxel-eluting balloon (Vesselin^®^). DCB, which must be at least 2 mm beyond the SB lesion distal to the injured segments, will be inflated for 60 s at a nominal pressure. The ratio of the DCB to SB diameter is between 0.8 and 1.0. After entering the human body, DCB should be delivered to the lesion within 2 min. After DCB angioplasty, two standard non-complaint balloons (MV and SB balloon diameter sized 1:1 according to vessel) will be used for further kissing inflation. A proximal optimization technique will be performed from the proximal stented segment to the carina level using a short non-complaint balloon (sized 1:1 according to the proximal MV).

Control group: Kissing inflation using two standard non-complaint balloons will be performed. The proximal optimization technique will be performed from the proximal stented segment to the carina level using a short non-complaint balloon (sized 1:1 according to the proximal MV).

If there is TIMI flow < 3 or ≥ type C dissection or residual stenosis > 30% in SB, a DES will be implanted in the SB by the two-stent technique.

### Intracoronary imaging and study stents

Intracoronary imaging tools (optical coherence tomography or intravascular ultrasound) will be selected by the interventional cardiologists. DES is used for all stent-implanted lesions.

### Study endpoints

#### Primary endpoint

LLL of SB at the 9-month follow-up will be assessed by quantitative coronary angiography (QCA) analysis. LLL is defined as the difference between the post-operative minimal lumen diameter (MLD) and the follow-up MLD.

#### Secondary endpoints

(1) Procedural success without clinical or ischemic events during the initial hospitalization, LLL of MV and binary angiographic restenosis in MV and SB at the 9-month follow-up by QCA.

(2) The proportion of patients with a final post-PCI quantitative flow ratio (QFR) result ≤ 0.80 for SB at the 9-month follow-up.

(3) Major adverse cardiac events (including cardiovascular death, target vessel-related myocardial infarction and ischemia-driven revascularization in MV and SB) during the 24-month follow-up.

Cardiac death, myocardial infarction and ischemia-driven revascularization are defined according to ARC-Academic Research Consortium guidelines (15). All endpoints will be reported on site using an electronic web-based capture system. All clinical events will be evaluated by an independent clinical events committee (CEC) unaware of the group assignment of the patients.

### Medications

All enrolled patients will receive dual antiplatelet therapy for at least 12 months in accordance with local practice and contemporary guidelines. It is recommended that patients take a loading dose aspirin (300 mg) and clopidogrel (300 mg) or ticagrelor (180 mg) at least 6 h before PCI. Heparin or alternative antithrombotic agents (such as bivalirudin) are used during PCI. After PCI, long-term use of 100 mg/day aspirin is recommended. The duration of treatment with 75 mg/day clopidogrel (or 90 mg/day ticagrelor twice) will be at least 12 months.

### Follow-up

Clinical follow-up by office visit or telephone contact is conducted at 30 days (± 7 days), 3 months (± 7 days), 6 months (± 7 days), 12 months (± 30 days), 18 months (± 30 days) and 24 months (± 30 days) by the enrolling site for outcome evaluation after discharge. Coronary angiography is performed at 9 months (± 7 days) by the enrolling site unless clinically indicated earlier.

### Quantitative coronary analysis

QCA analysis at baseline, intraoperative, postoperative, and follow-up will be conducted off-site in the core laboratory of Shaanxi Provincial People's Hospital. Images will be analyzed by two experienced interventional cardiologists unaware of the study design. Coronary angiography at baseline (preprocedure) and postprocedure of the target lesions must include at least 2 injections followed by intracoronary injections of nitroglycerin (recommended 100–200 μg, unless clinically contraindicated). A bifurcated view must be provided for all patients. There should be at least a 30° angular difference between the two baseline angiograms. Balloon dilatation and stent implantation during PCI are recorded by short cine runs.

### QFR assessment

QFR at postoperative and 9-month follow-up will be conducted off-site by two well-trained and blinded technicians in the core laboratory of Shaanxi Provincial People's Hospital by a QFR system software (AngioPlus, Pulse Medical Imaging Technology, Shanghai, China).

### Data collection and management

Researchers receive intensive training in the research requirements to improve the data quality and will be responsible for the data collection and entry. Data management will be performed by the core laboratory of Shaanxi Provincial People's Hospital. Researchers at each medical center will collect the data needed for this clinical study, including baseline clinical characteristics, medical treatments, laboratory results, interventional treatments, and outcomes. These text data will be entered into the Data Management System. All data will be transmitted anonymously. To ensure the traceability of all data, the collected data will be kept for at least 5 years. An independent data monitoring committee has been established and will check the accuracy of the data on a quarterly basis.

### Trial status

This clinical trial is ongoing. Participants are currently being recruited from 8 medical centers in China. The target number of subjects at each medical center is determined by the number of daily coronary angiography patients and the requests from each medical center.

### Sample size and statistical analyses

Since there is no previously published reference for similar research data, we hypothesized that the LLL of SB would be 0.25 ± 0.25 mm in the research group and 0.37 ± 0.40 mm in the control group based on the previous retrospective cases in our medical center. To achieve a power of 0.8 and a 2-sided critical threshold of 0.05, and considering a possible dropout rate of 20%, each group must contain a minimum of 70 patients. Therefore, this study requires 70 subjects in the research group and 70 subjects in the control group.

Statistical analysis will be performed by an independent core laboratory (CERC). Data for patients/lesions will be analyzed on the basis of this protocol. All statistical analyses will be performed with the statistical software package SPSS 24.0 (SPSS Inc., Chicago, USA). The normality of the distribution of continuous variables will be determined with the Kolmogorov–Smirnov test. Continuous variables will be expressed as the mean ± standard deviation (normal distribution) or the median and interquartile range (non-normal distribution). Categorical variables will be expressed as frequencies and percentages. Continuous variables will be compared by Student's *t-test* (normal distribution) or the Mann–Whitney *U* test (non-normal distribution). The chi-square test or Fisher's exact probability method will be applied for categorical data. Kaplan–Meier survival analysis will be used to generate survival curves with time-to-event data, and the log-rank test will be used for comparison. A Cox proportional hazard model with reporting HR and 95% CI will be used for comparisons between the two groups. All tests are two-sided, and a *P* value < 0.05 will be considered statistically significant.

### Ethical conduct

This clinical trial is conducted in accordance with the Declaration of Helsinki. The clinical trial protocol and written informed consent forms were reviewed and approved by the Clinical Trial Ethics Committee of Shaanxi Provincial People's Hospital (No. 2020-R008) and accepted by each medical center. Written informed consent is required from all enrolled patients. The results of this clinical trial will be published in a peer-reviewed journal.

## Discussion

Coronary bifurcation lesions are frequently encountered by interventional cardiologists. Compared to standard interventions, coronary bifurcation lesions are a challenging subset with higher technical complexity and worse outcomes (16). Coronary bifurcation is a complex anatomical structure consisting of the SB, proximal MV, and distal MV. Coronary bifurcation lesions are a complex subset of lesions due to several factors: plaque location, plaque burden, branch diameter, branch angle, bifurcation site and the presence of more than two branches. In addition, PCI of coronary bifurcation lesions may also cause anatomical changes (plaque displacement or dissection) (4, 17). These unique structural and bifurcation lesion characteristics can be altered and become even more complicated during and after PCI (18). Therefore, it is crucial to select the most appropriate strategy and technique for coronary bifurcation lesions.

The optimal interventional method for coronary bifurcation lesions has been a matter of considerable controversy over the past few years. DES helps to significantly decrease the risk of target vessel revascularization and restenosis. However, two-stent technique in some bifurcation lesions will result in substantial metal residues in the lumen, which is particularly relevant to stent thrombosis and in-stent restenosis (19). In fact, SB stents may cause insufficient coverage of the SB ostium or excessive strut protrusion into the MV (20). The European Bifurcation Club (EBC) recommends that PCI adhere to the principle of “keep it simple and safe” in the selection of stents for bifurcation lesions and to try to limit the number of stents as much as possible (21). Currently, the provisional stenting technique of implanting one stent in MV and ignoring SB lesions (unless the clinical situation requires implantation of one stent in SB) is considered a preferable method for most bifurcation lesions (1). However, regardless of whether there is a final kissing balloon inflation at the end, approximately 17–19% of cases still undergo SB restenosis (6). Therefore, the dilemma of how to reduce SB restenosis still exists, and further research on strategies that help to reduce restenosis for SB is necessary.

In the past few years, DCB has proven its clinical safety and effectiveness for coronary artery small vessels and in-stent restenosis, without the need for permanent implantation of metallic struts. The use of an MV stent first following a DCB in SB is an attractive perspective according to the “keep it simple and safe” principle for coronary bifurcation lesions (21, 22). First, DCB can reduce the complexity of the PCI procedure, as the two-stent technique requires extensive technical steps. Second, DCB can significantly increase the incidence of provisional stenting techniques and reduce the incidence of device-related failures (stent thrombosis and in-stent restenosis) associated with two-stent technique. Moreover, compared with conventional dilation, DCB with antiproliferative agents can provide better outcomes for SB, mitigating the limitations of plain angioplasty (5). These aspects represent the rationale for the predicted efficacy and safety of DCB for SB of coronary bifurcation lesions.

The BIOLUX-I study aimed to evaluate the feasibility of a DES in MV and a paclitaxel-eluting balloon in SB of coronary bifurcation lesions (10). Thirty-five patients were enrolled with a 12-month follow-up. The results suggested that the LLL in SB was 0.10 ± 0.43 mm without binary restenosis at the 9-month angiographic follow-up. The total incidence of major adverse cardiac events was 5.9%, and target lesion revascularization was 2.9% without stent thrombosis at the 12-month clinical follow-up. The major limitations of this study were the exclusion of unprotected left main target lesions and very proximal coronary bifurcation lesions (target lesion within 5 mm of the origin of the right coronary artery, left circumflex and left anterior descending).

The DEBSIDE study enrolled 52 patients with coronary bifurcation lesions. The patient received DCB inflation in SB after the systematic implantation of DES in MV. The results showed that the LLL in SB was −0.04±0.34 mm at the 6-month angiographic follow-up (11). Therefore, a DES in MV and a DCB in SB seem to be safe and effective with a low LLL. Although there is no control group and the number of patients recruited was low, the results provided by the above two trials show that a DES in MV and a DCB in SB have good performance for coronary bifurcation lesions.

As a complement to a provisional stenting strategy, DCB remains very attractive for bifurcation lesion management. However, many questions, including selection for SB, technology (DCB with or without final kissing balloon or proximal optimization technology), and the actual impact on clinical endpoints remain unanswered (5, 23). To date, studies exploring the efficacy of DCB in *de novo* coronary bifurcation lesions have many limitations, and no conclusive evidence has been provided. Therefore, larger and more consistent studies are required to assess the efficacy and safety of DCB in this subset.

Cutting balloon is a non-compliant balloon with longitudinally bonded microtomes on the surface for cutting atherosclerotic plaques, which can facilitate plaque extension, minimize intimal injury, reduce elastic recoil, prevent progressive dissections, and facilitate stent delivery (13). Deploying a cutting balloon in SB can effectively prevent plaque displacement or plaque protruding into MV, which can reduce the incidence of SB compromise and restenosis (13). Compared with cutting balloon angioplasty, the combination of DCB and cutting balloon for SB in coronary bifurcation lesions may be more effective and safer. However, to the best of our knowledge, there is no randomized comparison of DCB combined with cutting balloon vs. cutting balloon angioplasty for SB after a DES in MV of true coronary bifurcation lesions, making the ongoing gaps in knowledge a relevant issue.

## Conclusion

The optimal treatment of SB in coronary bifurcation lesions is still controversial. A DES in MV remains the default strategy for most coronary bifurcation lesions that can be managed with a provisional stenting strategy. DCB combined with cutting balloon in SB might improve the short- and long-term outcomes of bifurcation lesions. This study is the first multicenter, prospective, randomized controlled trial to test the hypothesis that DCB combined with cutting balloon is superior to cutting balloon angioplasty for SB after a MV stent implantation in true coronary bifurcation lesions. This trial will provide evidence to establish methods for SB in coronary bifurcation lesions.

## Ethics statement

The studies involving human participants were reviewed and approved by Clinical Trial Ethics Committee of Shaanxi Provincial People's Hospital. The patients/participants provided their written informed consent to participate in this study.

## Author contributions

HW and XS contributed to the conception and design of the study. HW wrote the manuscript. JD, WH, and HC contributed to data collection. LL, XL, and XY managed the clinical practice organization. All authors contributed to refinement of this study protocol and approved the submitted version.

## Funding

This study is supported by the Research and Development Program of Shaanxi Province (No. 2022ZDLSF02-03) and the Science and Technology Talent Support Program of Shaanxi Provincial People's Hospital (No. 2021LJ-09).

## Conflict of interest

The authors declare that the research was conducted in the absence of any commercial or financial relationships that could be construed as a potential conflict of interest.

## Publisher's note

All claims expressed in this article are solely those of the authors and do not necessarily represent those of their affiliated organizations, or those of the publisher, the editors and the reviewers. Any product that may be evaluated in this article, or claim that may be made by its manufacturer, is not guaranteed or endorsed by the publisher.
